# Engineering Cell–ECM–Material Interactions for Musculoskeletal Regeneration

**DOI:** 10.3390/bioengineering10040453

**Published:** 2023-04-07

**Authors:** Calvin L. Jones, Brian T. Penney, Sophia K. Theodossiou

**Affiliations:** Department of Mechanical and Biomedical Engineering, Boise State University, 1910 University Dr MS2085, Boise, ID 83725, USA

**Keywords:** extracellular matrix (ECM), musculoskeletal tissue engineering, musculoskeletal regeneration, skeletal muscle, tendon, cartilage, bone, atomic force microscopy (AFM)

## Abstract

The extracellular microenvironment regulates many of the mechanical and biochemical cues that direct musculoskeletal development and are involved in musculoskeletal disease. The extracellular matrix (ECM) is a main component of this microenvironment. Tissue engineered approaches towards regenerating muscle, cartilage, tendon, and bone target the ECM because it supplies critical signals for regenerating musculoskeletal tissues. Engineered ECM–material scaffolds that mimic key mechanical and biochemical components of the ECM are of particular interest in musculoskeletal tissue engineering. Such materials are biocompatible, can be fabricated to have desirable mechanical and biochemical properties, and can be further chemically or genetically modified to support cell differentiation or halt degenerative disease progression. In this review, we survey how engineered approaches using natural and ECM-derived materials and scaffold systems can harness the unique characteristics of the ECM to support musculoskeletal tissue regeneration, with a focus on skeletal muscle, cartilage, tendon, and bone. We summarize the strengths of current approaches and look towards a future of materials and culture systems with engineered and highly tailored cell–ECM–material interactions to drive musculoskeletal tissue restoration. The works highlighted in this review strongly support the continued exploration of ECM and other engineered materials as tools to control cell fate and make large-scale musculoskeletal regeneration a reality.

## 1. Introduction

Engineering cell-extracellular matrix (ECM) interactions to achieve desired regenerative outcomes are promising approaches in musculoskeletal tissue engineering. Musculoskeletal tissue injuries are a common and increasing clinical problem in the US and around the world [1]. Injuries and diseases of the skeletal muscles, tendons, cartilage, and bones have widespread impacts on the activities and quality of daily life, as musculoskeletal tissues enable movement and daily function. Current musculoskeletal injury treatments rely on rehabilitative or surgical interventions that may not restore normal function, resulting in long-term disability, with associated economic losses in terms of lost wages, decreased productivity, and high healthcare costs [1]. Regenerative medicine promises to improve the prognosis for musculoskeletal pathologies. The last fifteen years have substantially deepened the knowledge of how cells and biomaterial scaffolds can restore functionality to musculoskeletal tissues [2]. In this review, we highlight recent and noteworthy studies that have engineered the relationship between cells, the ECM, and natural or synthetic biomaterials to selectively exploit desired interactions with musculoskeletal cells and tissues. 

Engineering the ECM to facilitate desired cell–cell and cell–material interactions is a relatively new approach in tissue engineering. In the last decade, biomaterial fabrication methods have progressed to enable precise control over the mechanical, chemical, and biological properties of the extracellular microenvironment [3]. Thanks to this enhanced control of scaffold properties, tailoring the microenvironment to support targeted cell growth and differentiation is a viable tissue engineering strategy. Scaffolds with highly controlled porosity [4,5], pH-responsiveness [6,7], stiffness [8,9,10,11], and biochemistry [12] are increasingly used to drive cellular behavior towards the regeneration of large-scale musculoskeletal tissues and musculoskeletal tissue interfaces [13,14,15]. Additional components of the extracellular environment, such as growth factors and extracellular vesicles, also direct cell behavior, often in combination with the ECM; engineered approaches focusing on these aspects of the microenvironment are discussed elsewhere [16]. In this review, we focus on approaches targeting or utilizing the ECM, though we note that several of the highlighted studies incorporate extracellular vesicles and growth factors alongside their ECM focus.

The cell- and tissue-derived extracellular matrix (ECM) has been extensively characterized for uses in tissue engineering, and it has been reviewed elsewhere [2,17]. To generate ECM for scaffolds, various methods have been developed for extracting ECM from human [18,19] and animal [20,21,22] tissues. A persistent concern is the rejection of animal ECM-containing scaffolds in human recipients, though this review highlights studies that indicate that the animal ECM can be used safely and successfully in human patients [23]. Human-derived ECM, such as ECM generated from human acellular dermal matrix, also has potential uses in bioscaffolds that enhance musculoskeletal regeneration [24]. Human-sourced ECM has recently been isolated from the decellularized human chorion matrix [19], transforming the placenta from an organ that is commonly discarded as medical waste to a rich source of ECM starting material for tissue engineering applications in several tissues, including the musculoskeletal system. Moving forward, genetic engineering techniques, such as CRISP-Cas9, may expand the potential uses of animal-derived ECM in humans. Additionally, though it is less common than ECM originating from animal or human tissues, cell-derived ECM has been generated from cells cultured for the purpose of producing ECM with specific, desirable qualities, with varied but consistent success [2,25]. In the concluding sections of this review, we briefly discuss how genetic engineering may enable the generation of highly specialized and useful ECM from genetically-modified cells possessing desirable ECM characteristics. We predict that ECM and ECM-derived biomaterials will continue to be valuable tools for directing and engineering cell functions. 

The value of engineering the ECM to foster specific cell–ECM interactions extends beyond its use in regenerative approaches and into its widespread potential applications in directing cell function throughout entire tissue and organ systems [26]. Engineering the ECM to resemble various musculoskeletal pathologies can be used to model injury and disease, enhancing the search for treatments and cures. To treat musculoskeletal disease, a deeper understanding of the pathologies is needed. Engineering the ECM to resemble specific conditions, or to mimic particular stages of development or aging, has numerous uses in musculoskeletal research. Throughout this review, we use the term “engineering” in reference to the ECM to denote research approaches that extract, augment, modify, pattern, or otherwise adapt features of the ECM and purposefully impact cell- or tissue-level experimental outcomes. We pay special attention to emerging treatments for musculoskeletal fibrosis, a persistent ECM-mediated problem both within and outside the musculoskeletal system. Our goal is to illustrate promising ideas in the hopes of fostering novel discussions and collaborations that move the field of musculoskeletal tissue engineering forward.

## 2. Survey of Engineered Cell–ECM–Material Interactions in Musculoskeletal Tissues

### 2.1. Skeletal Muscle

Selected approaches utilizing cell–ECM–material interactions to regenerate skeletal muscle are summarized in Table 1. Skeletal muscle composes nearly 40% of the weight of the average human body [27], enables locomotion and fine motor skills, and participates in multiple homeostatic processes [28]. Skeletal muscle ECM supports the function of muscle cells by providing three-dimensional scaffolding for their function and growth, as well as facilitating interactions with the various collagens, glycoproteins, proteoglycans, and elastins that give skeletal muscleits mechanical and biological properties [29]. The ECM regulates skeletal muscle growth, maintenance, response to injury, and repair. Importantly, the structure of skeletal muscle ECM directly contributes to the tissue’s mechanical capabilities.

The highly organized, hierarchical structure of skeletal muscle has made it a particularly challenging tissue to engineer, as its structure provides unique mechanical properties. Replicating muscle structure and the complex multi-tissue interfaces required for muscle function, such as neuromuscular junctions (NMJs) and tendons, has proven elusive. While muscle cells from human and non-human sources readily grow and differentiate in vitro, and they can be successfully implanted in numerous animal models, the resulting muscle tissue often lacks the force production, growth, and vascularization capacities of native muscle. A significant limitation of several regenerative approaches for restoring skeletal muscle is the inability to replicate the structural and spatial components of healthy muscle tissue. This problem is especially pronounced in research attempting to reverse volumetric muscle loss (VML) [30]. VML results from extensive trauma to the muscles that overwhelms their intrinsic regenerative capacity [31]. VML is characterized by a complete breakdown of the muscle ECM and its associated structures [31]. Restoring that structure in lab-grown muscle may be the key to addressing not only VML, but other traumatic and wasting diseases characterized by widespread muscle tissue damage.

#### 2.1.1. ECM-Based Approaches for Skeletal Muscle Regeneration

**Figure 1:**
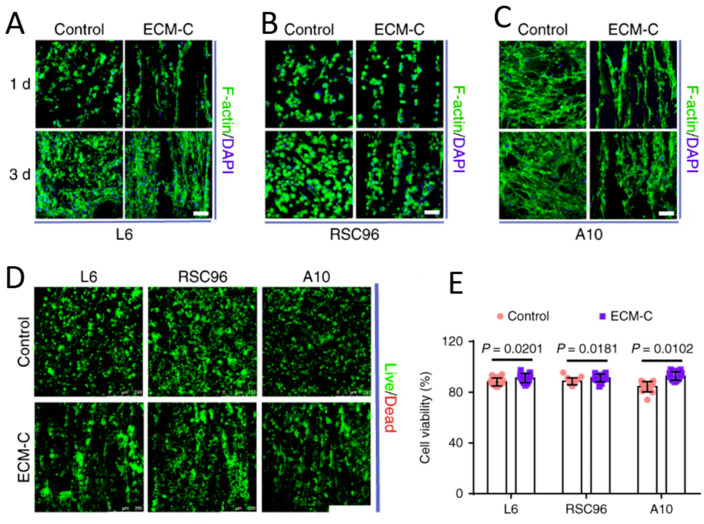
Materials composed of cells and ECM show promise for recreating the complex, hierarchical structure of functional skeletal muscle tissue. ECM–cell composite scaffolds effectively guide cells towards the spatial organization required for muscle, nerve, and blood vessel formation. (**A**–**C**) Cellular guiding effects of ECM–cellular (ECM–C) and control scaffolds. Skeletal actin fibers and nuclei of (**A**) L6, (**B**) RSC96, and (**C**) A10 cells were respectively stained with fluorescein isothiocyanate (FITC) conjugated phalloidin and DAPI at one and three days. (**D**,**E**) Live/dead staining and cell viability of L6, RSC96, and A10 cells on different scaffolds at seven days. Live cells are stained green and dead cells are stained red (*n*  =  15). Figure adapted with permission from Zhu et al. 2019 [33].

Engineered approaches replicating the organized ECM of muscle have seen some success in terms of the force production of the regenerated muscle [32] and have improved the formation of NMJs. The restoration of highly oriented and structured muscle and NMJ tissue may require engineering the ECM with appropriate templates or sacrificial scaffolds until cells can differentiate and deposit their own matrix. Sacrificial templates enhanced ECM deposition in a rat model of a tibialis anterior defect, where engineered ECM scaffolds with parallel microchannels were subcutaneously implanted, and the template was then removed and decellularized (Figure 1). Compared to controls, rats that received the scaffolds had extensive neotissue formation in the grafting area, as well as cell infiltration, blood vessel formation, and new ECM deposition [33]. Neo-muscle tissue had acetylcholine receptors and nerve fiber contacts resembling early NMJ formation, suggesting this engineered ECM microchannel model is a promising in vitro and in vivo platform.

Although decellularized ECM has been shown to aid tissue regeneration, it may require additional materials for effective tissue delivery. A recent study coupled the use of ECM with extracellular vesicles (EVs), cell-secreted nanoparticles that convey intercellular signals to propagate tissue repair. Decellularized muscle ECM was combined with human EVs derived from Wharton Jelly mesenchymal stromal cells (MSC EVs) to boost tissue regeneration in a murine VML model [34]. Thirty days after the removal of the tibialis anterior, ECM plus MSC EV treated mice had significantly higher myosin heavy chain (MHC) expression compared to controls treated with muscle ECM in phosphate-buffered saline (PBS). Notably, the EV-treated mice showed gains in muscle function compared to the control group, underscoring the potential of ECM and EVs as aids in tissue-specific regeneration and functional restoration.

Xenogeneic ECM (ECM derived from animal tissue) scaffolds were tested as platforms for skeletal muscle restoration in another complex injury model [35]. A scaffold composed of small intestinal submucosa ECM (SIS-ECM) was used to repair resections in canine vastus lateralis and vastus medialis muscles. At 1, 2, 3, and 6 months post-injury, muscle function was not restored [35]. However, the SIS–ECM appeared to promote integration of soft and bony tissues, suggesting it may be a useful tool in engineering the ECM after injury to promote an integrative cellular response. Other uses of xenogeneic ECM in skeletal muscle tissue engineering have involved porcine bladder tissue in murine models of VML, and they have shown improved perivascular stem cell mobilization and accumulation, with increased skeletal muscle cell proliferation at the injury sites [23]. The porcine bladder ECM supported the formation of stimulus-responsive skeletal muscle cells and tissues in mice, which resulted in functional improvement in three implanted human patients. Notably, all patients showed signs of de novo formation of muscle tissue and vasculature. In the same study, an accompanying murine VML model found no evidence of new skeletal muscle tissue proliferation in the untreated controls. Conversely, animals treated with ECM formed islands of desmin and myosin heavy chain-positive (MHC+) striated skeletal muscle throughout the defect and scaffold placement areas, consistent with an ECM-mediated constructive remodeling effect [23]. EMG analysis further showed that ECM treated mice displayed muscle activation characteristics closer to those of uninjured mice, rather than untreated controls. Taken together, these studies suggest xenogeneic ECM has the potential to improve functional muscle regeneration in human patients. 

#### 2.1.2. ECM-Based Personalized Disease Models

Engineered muscle ECM is a crucial component of recently developed and highly accurate in vitro disease models, which are of significant interest as personalized test platforms for novel muscle-targeting therapeutics. Collagen VI- related dystrophies (COL6-RDs) are rare congenital neuromuscular dystrophies where alterations in one of the three collagen VI genes change the collagen’s incorporation into the ECM, severely impacting the mechanical coherence of the matrix [36]. COL6-RDs result in muscle weakness, proximal joint contractures, and distal hyperlaxity. To bridge the gap between the specific ECM features of COL6-RD patients and the known clinical phenotype, recent work engineered a novel, personalized preclinical model of COL6-RDs using cell-derived matrices (CDMs). These CDMs were developed using the forearm skin fibroblasts of patients with COL6-RD and healthy donors. Disease markers were significantly increased in CDMs from COL6-RD patients compared to controls (CDMs derived from healthy patients). Additionally, higher Collagen VI and fibronectin alignment, length, width, and straightness were observed in control CDMs compared to patient-derived CDMs, suggesting the model successfully captured the matrix characteristics of COL6-RD [36]. This and other studies summarized elsewhere [37] hint towards a future of personalized disease models for identifying the most effective treatment for individual patients. Engineering the ECM to accurately replicate specific disease morphologies will lay the groundwork for novel surgical, rehabilitative, and pharmacological treatments of currently incurable conditions.

The future of engineered cell–ECM interactions in skeletal muscle tissue engineering extends to personalized injury models. ECM–cell materials can potentially recreate the tissue structure that is destroyed during VML, with the goal of restoring the large quantities of the specific muscle tissue (for example, the quadriceps or the gastrocnemius) lost in VML, thus reversing the associated disability. Recreating the highly organized mechanical and biological structure of muscle tissue remains a significant challenge. Both natural and engineered ECM, as a standalone biomaterial or in combination with other scaffolds, is uniquely suited to providing a culture environment that mimics native muscle. Though much exploration is still needed regarding the role of ECM in directing cell behavior in degenerative or muscle wasting diseases, improved in vitro and in vivo models of engineered ECM–cell interactions will advance our knowledge of which mechanical, biochemical, and genetic factors synergize to initiate skeletal muscle pathologies. 

To prevent or reverse skeletal muscle atrophy, a viable strategy may be targeted hypertrophy using ECM-based materials to stimulate muscle cells. Matricellular proteins play key roles in ECM–cell communication and are necessary to understand in engineering ECM–cell interactions for musculoskeletal regeneration. A recent study indicated that matricellular protein cellular communication network factor 2 (CCN2), also known as connective tissue growth factor (CTGF), is induced during a time course of overload-driven skeletal muscle hypertrophy in mice and may be a useful addition to current strategies. Genetically engineered mouse models for myofiber-specific CCN2 gain- and loss-of-function were subjected to mechanical stimuli via muscle overload. Testing showed that myofiber-specific deletion of CCN2 blunted the muscle’s hypertrophic response to overload without interfering with ECM deposition. Conversely, CCN2 overexpression was efficient in promoting overload-induced aberrant ECM accumulation without affecting myofiber growth [38]. The study results, following these genetic alterations, suggest the existence of independent ECM and myofiber stress adaptation reposes, both of which appear to be mediated by CCN2. Furthermore, the study proposed that CCN2 acts by regulating focal adhesion kinase (FAK)-mediated transduction of overload-induced extracellular signals, including interleukin 6 (IL6), as well as their downstream impact on global protein synthesis in skeletal muscle [38]. These findings highlight the crucial role of the muscle-derived ECM factor CCN2 in enabling mechanically induced, hypertrophic muscle growth, and they suggest that providing native CCN2 may be a significant advantage of ECM-based regenerative approaches.

**Table 1:** Recent and noteworthy studies focused on cell–ECM–material interactions in skeletal muscle.

Tissue	Model	Material	Main Findings
Skeletal Muscle	ECM combined with extracellular vesicles (EVs) and mesenchymal stem cells (MSCs) in a murine VML model.	Decellularized ECM and extracellular vesicles (EVs).	Muscle regeneration was enhanced after 30 days in mice treated with ECM and EVs. Higher MHC and gains in muscle function compared to control groups [34].
	ECM scaffolds with parallel microchannels (ECM-C) by subcutaneous implantation of sacrificial templates followed by template removal and decellularization.	ECM scaffolds with parallel microchannels (ECM-C).	Compared to controls, rats that received the scaffolds had extensive neo-tissue formation in the grafting area, as well as cell infiltration, blood vessel formation, and new ECM deposition, which were not observed in the controls. Neo-muscle tissue had acetylcholine receptors and nerve fiber contacts, resembling early neuromuscular junction formation [33].
	Unilateral resection of the distal third of the vastus lateralis and medial half of the distal third of the vastus medialis in dogs; defects replaced with scaffolds composed of small intestinal submucosa extracellular matrix (SIS-ECM).	Scaffolds composed of small intestinal submucosa extracellular matrix (SIS-ECM).	SIS-ECM promoted integration of soft and bony tissues, suggesting it may be a useful tool in engineering the ECM after injury to promote an integrative response in the cells [35].
	Xenogeneic porcine urinary bladder ECM scaffolds used as a surgical treatment for volumetric muscle loss in both a preclinical rodent model and human male patients.	Xenogeneic porcine urinary bladder ECM Scaffolds.	Porcine bladder ECM supported the formation of stimulus-responsive skeletal muscle cells and tissues in mice, and functional improvement was observed in three implanted human patients. ECM-treated mice showed muscle activation [23].
	Preclinical model of collagen VI- related dystrophies (COL6-RDs) using cell-derived matrices (CDMs) developed using the forearm skin fibroblasts of both patients with (COL6-RD), as well as from healthy donors without neuromuscular disease.	Cell-derived matrices (CDMs) developed using the forearm skin fibroblasts of both patients with (COL6-RD), and from healthy donors without neuromuscular disease.	Disease markers were significantly increased in CDMs from COL6-RD patients compared to controls (CDMs derived from healthy patients). Higher collagen VI and fibronectin alignment, length, width, and straightness were observed in control CDMs compared to patient-derived CDMs [36].
	Decellularized canine placentas and murine skeletal muscle ECM placed in male Wistar rats with pockets at the posterior limbs.	Decellularized canine placentas and murine skeletal muscles.	Higher percentage of proliferative PCNA+ cells three days after implantation in placenta-derived matrices, compared to muscle derived matrices. Higher percentage of CD163^+high^ macrophages in muscle-derived ECM; higher percentage of CD163^+low^ macrophages found in placenta-derived ECM 3- and 15-days post-implantation [39].

#### 2.1.3. Interactions between ECM and the Immune System in Skeletal Muscle

Immune cell–ECM interactions are also critical for muscle growth and regeneration. Despite their emerging potential in regenerative approaches, decellularized allogeneic and xenogeneic ECM scaffolds may cause an aggressive inflammatory response when implanted. To combat this, a recent study compared decellularized canine placentas and murine skeletal muscles as scaffolds for regenerating skeletal muscles in a rat model [39]. Muscle pockets were created at the posterior limbs of male Wistar rats, into which the muscle and placenta derived ECM were implanted. Three days after implantation, a higher percentage of proliferative cells (PCNA^+^) was seen in placenta-derived matrices compared to muscle derived matrices [39]. Additionally, higher percentages of CD163^+high^ macrophages were observed in the muscle derived ECM group, whereas CD163^+low^ macrophages were found at higher percentages in the placenta-derived ECM group three- and fifteen-days post implantation. These findings indicate that the local inflammatory response mediated by the implantation of the placenta-derived ECM was similar to that of the allogeneic muscle ECM, suggesting that placenta-derived ECM may minimize inflammation. Placental tissue is an abundant biomaterial that is often discarded as medical waste. Deriving ECM scaffolds from the placenta is desirable, as it is easier to procure than skeletal muscle, and decellularization produces large quantities of tissue. Future decellularized ECM approaches may source placental and other currently underutilized tissues, as research showing the source of the ECM to be consequential continues to emerge. 

Decellularized ECM-based approaches may overstimulate the immune system by using excessive ECM material. Insufficient ECM can compromise musculoskeletal tissue function, but excessive ECM deposition that mimics or instigates fibrosis is detrimental, as it drives replacement of the resident muscle fibers with collagenous scar tissue, leading to atrophy and poor mechanical function. Unwanted infiltration of fibrotic tissue is commonly seen in implantation of allogeneic ECM scaffolds and in acute surgical injury. Anticipation and prevention of the physiological mechanisms responsible for fibrosis are needed within regenerative approaches. To this end, a recent study targeted cyclooxygenase-2 (COX-2), the rate-limiting enzyme in synthesis of prostaglandin and a positive regulator in pathophysiological processes, such as inflammation and oxidative stress [40]. Injured muscles in both human patients and mouse models overexpressed COX-2 compared to non-damaged muscle regions. COX-2 was also upregulated in human and murine fibroblasts following TGF-β stimulation [40]. The same study investigated how celecoxib, a COX-2 inhibitor, impacted fibrogenesis in human patients. Celecoxib-mediated COX-2 inhibition was anti-fibrotic via inhibition of fibroblast differentiation, proliferation, and migration, as well as inactivation of TGF-β-dependent signaling, non-canonical TGF-β pathways, and suppression of both reactive oxygen species (ROS) formation and oxidative stress [40]. Similarly, celecoxib-mediated inhibition of COX-2 in mouse models resulted in decreased tissue fibrosis and increased skeletal muscle fiber preservation, reflected by less ECM formation and myofibroblast accumulation with decreased p-ERK1/2, p-Smad2/3, TGF-βR1, VEGF, NOX2, and NOX4 expression. This work suggests that pharmacologically targeting the COX-2/PDK1/AKT signaling pathway may be a beneficial co-intervention to block immune rejections when using ECM scaffolds in skeletal muscle.

#### 2.1.4. Other ECM-Based Approaches in Skeletal Muscle

Other ECM proteins, such as SPARCL1, have recently been targeted due to their roles in myogenic differentiation. SPARCL1 was quantified in transfected C2C12 mouse muscle myoblasts and in a mouse tibialis anterior injury model, where in vivo results suggested that SPARCL1 is associated with muscle damage repair in mice, and in vitro data showed that SPARCL1 binds to bone morphogenetic protein 7 (BMP7) by regulating BMP and transforming growth factor (TGF)-β cell signaling [41], both pathways that ultimately promote myogenic differentiation in C2C12 cells. Using CRISPR/Cas9, the same study showed that SPARCL1 activated BMP/TGF-β to promote the differentiation of C2C12 cells, and BMP7 molecules interacted directly with SPARCL1. Overall, SPARCL1 influenced the expression of BMP7 and activity of the BMP/TGF-β signaling pathway, while SPARCL1 activation was accompanied by BMP7 inhibition in C2C12 cells, confirming that SPARCL1 affects BMP7 expression and can promote C2C12 cell differentiation through the BMP/TGF-β pathway. 

In addition to signals from ECM proteins, muscle cell differentiation is dependent on the correct assembly of the ECM itself, which is in turn regulated by matrix-metalloproteinases (MMPs). Reversion-inducing-cysteine-rich protein with kazal motifs (RECK) is a membrane-anchored protein that negatively regulates the activity of different MMPs. RECK’s role in skeletal muscle differentiation, regeneration, and fibrosis have only recently been explored, but one study showed that, during myogenic differentiation of C2C12 myoblasts and satellite cells on isolated muscle fibers, RECK was transiently upregulated, and upregulation alternated with periods of RECK reduction [42]. Additionally, when RECK levels were reduced, C2C12 myoblasts were likely to enter their differentiation program with an accelerated differentiation process. RECK- deficient (RECK±) mice models were also compared to WT controls. In vivo results indicated that the transient upregulation of RECK occurs during skeletal muscle regeneration and is accelerated in RECK± mice. The RECK± mice had diminished fibrosis in response to chronic muscle damage compared to WT controls, suggesting that RECK acts as a myogenic repressor during muscle formation and regeneration [42]. Attenuating the effects of RECK in the ECM may prove useful for engineered and regenerative muscle therapies. 

#### 2.1.5. ECM-Cell Interactions and Muscle Fibrosis

Beyond acute injuries, muscle fibrosis can often result from muscular dystrophies. Though there are many different pathologies that directly lead to muscular dystrophy, research focus has recently shifted to patients with defects in fukutin-related protein (FKRP). FKRP is a glycosyltransferase, with the only identified function of transferring ribitol-5-phosphate to α-dystroglycan (α-DG) [43]. Interestingly, this modification is crucial for ECM attachment. A recent study showed that FKRP directs sialylation of fibronectin, a process essential for collagen recruitment to the muscle basement membrane [43]. The study findings indicated that FKRP regulates the major muscle–ECM linkages essential for fiber survival, and thus warrants further investigation as a disease axis for several muscular dystrophies. Understanding how the FKRP-dependent glycosylation of fibronectin regulates muscle pathology may prove useful, not only for understanding muscular dystrophies, but also as a new pathway to explore in ECM-mediated muscle regeneration. 

A related study specifically targeted Duchenne muscular dystrophy (DMD), which is characterized by increased muscle stiffness alongside a buildup of collagenous fibrotic tissue and other ECM materials. It was previously unknown if, and by which mechanisms, collagen organization changes with the progression of DMD in diaphragm muscle tissue, and it was difficult to predict how collagen organization influences the mechanical properties of the ECM. C57BL/10ScSn-Dmdmdx/J (mdx) and wild type (WT) mice models were compared and assessed for collagen fibers straightness and alignment after three months and six months. Collagen was significantly straighter and more aligned in the WT mice at both timepoints, though collagen fibers retained a transverse orientation relative to the muscle fibers in both models. Image-based finite element mechanical models predicted an increase in the transverse relative to longitudinal (muscle fiber direction) stiffness, with the stiffness ratio (transverse/longitudinal) increased in the mdx model compared to the WT at three and six months. This study highlights that the changes in diaphragm ECM structure and mechanical properties during the disease progression in the mdx muscular dystrophy mouse may be the underlying mechanism driving changes to the diaphragm muscle function that are severe complications of DMD. These results suggest that exploiting cell–ECM–material interactions to increase collagen fiber organization and alignment may be a viable strategy for engineering muscle grafts or materials to treat dystrophies and alleviate the ECM disorganization that fibrosis causes. 

Muscular fibrosis can also be the result of other pathologies, such as denervation (denervation-induced fibrosis), which follows loss of nervous system stimulation or connectivity to a skeletal muscle. A recent study showed that mice subjected to a unilateral sciatic nerve transection accumulated ECM protein, such as collagen and fibronectin, in the denervated hindlimb, together with increased levels of the profibrotic factors TGF-β and connective tissue growth factor (CTGF/CCN2). Mice that were hemizygous for CTGF/CCN2 or mice treated with a blocking antibody against CTGF/CCN2 had a reduced accumulation of ECM proteins after denervation, compared to control mice. Additionally, these mice had no changes in fibro/adipogenic progenitors (FAPs). The same study found that ECM proteins and CTGF/CCN2 levels were increased two to four days after denervation; however, TGF-β signaling did not increase until one to two weeks post-denervation. Blocking TGF-β did not decrease fibronectin or CTGF levels four days following denervation, suggesting that CTGF/CCN2 is not upregulated by canonical TGF-β signaling early after denervation. Further investigation is necessary to understand the other factors involved in the early fibrotic response following skeletal muscle denervation. Understanding these signals can lead to strategic ECM-mediated approaches to simultaneously regenerate muscle and reduce fibrosis in denervated areas. 

Primary myelofibrosis (PMF) is a type of myeloproliferative neoplasm (MPN) characterized by a buildup of fibrotic tissue in the bone marrow. Given the negative side effects of current treatment options, such as the JAK2 inhibitor, as well as ruxolitinib, which also does not always have high efficacy, recent research has turned toward the possibility that ECM and bone marrow (BM) microenvironments may have an important role in the development of PMF. Lysyl oxidase (LOX), an enzyme that plays a key role in the ECM by facilitating the cross-linking of collagen and elastin fibers, has recently been targeted as an area for research, as it has been shown to be upregulated in megakaryocytes (MKs) of PMF mice and in PMF patients. This upregulation alludes to the possibility that LOX has a role in the progression of BM fibrosis. As such, LOX has been identified as a potential novel therapeutic target for PMF, leading to the development of small molecule LOX inhibitors, PXS-LOX 1 and PXS-LOX 2, which have shown that they may slow the progression of PMF in preclinical studies. Furthermore, these inhibitors prove to be potential PMF therapeutic agents, as they can target the dysregulation of the ECM via LOX inhibition. Including LOX inhibitors into ECM-derived tissue engineering may be useful in preventing potential osteo-fibrotic diseases, such as PMF.

The ECM ultimately provides two avenues for guiding myogenic cell behavior: ECM–material interactions for improved scaffold fabrication, as well as ECM–cell interactions for enhanced control of cellular outcomes. Understanding ECM proteins and their role in cellular communication is proving to be a crucial element in EMC-mediated muscle regeneration. New materials that incorporate native or engineered ECM, or that mimic key aspects of the ECM, are supporting unprecedented control over cell fate following cell–material interactions. As myogenic materials continue to improve in terms of cost, reproducibility, and immunogenicity prevention, we expect that their uses and relevance in both clinical and research settings will expand. We now shift our focus to cell–ECM–material interactions in cartilage tissue engineering.

### 2.2. Cartilage

Selected approaches utilizing cell–ECM–material interactions to regenerate cartilage are summarized in Table 2. Cartilage tissue engineering aims to slow, halt, or reverse the progression of osteoarthritis (OA), an inflammatory disease that impacts daily function, and it is likely to affect over 75 million Americans and over 300 million patients globally by 2030 [1,44]. Changes to the body’s biochemical and mechanical environment initiate and drive OA. The ECM is critical in the context of OA, as alterations in ECM mechanics, particularly stiffness, have been shown to activate macrophages (MFs) [45,46]. Briefly, OA is associated with immune cell activation, including MFs [47], in response to stiffness changes in the ECM [48,49]. Further, ECM stiffening with alterations to the chondrocytes (cartilage cells) occurs in articular cartilage during OA [50,51,52,53]. In a recent proof-of-concept study, the ECM mechanical properties were tuned to reduce pro-inflammatory macrophage phenotypes, highlighting the therapeutic potential of keeping ECM mechanics within a desired stiffness range [46]. Given the lack of treatments that can restore normal articular function, once clinically-identifiable OA sets in, as well as the early promise of mechanics-based anti-inflammatory interventions in engineered cartilage ECM [45], and there is significant interest in engineering articular cartilage ECM with mechanical and biochemical properties that can treat or reverse OA.

Currently, invasive and noninvasive methods are used to treat early to moderate OA. Available treatments, including pharmaceuticals and physical therapy, do not adequately address the problem of tissue degeneration, but instead reduce pain and slow OA progression. However, existing treatments do not meaningfully improve the underlying clinical problem of tissue deterioration. A recently developed method that directly addresses cartilage damage is targeted cellular therapy. While its promising results are reviewed elsewhere [54], a key takeaway from approaches utilizing targeted cell delivery is that is that chondrocytes can undergo rapid dedifferentiation and lose their ability to produce hyaline cartilage and form fibrocartilage during in vitro culture, likely due to the lack of ECM-supplied spatial and mechanical cues. Engineered cartilage ECM that can provide chondrocytes with the necessary signals is an important and ongoing area within OA research.

Changes in the nanoscale structure of cartilage ECM lead to changes in macroscale tissue behavior that may initiate and propagate OA, but these changes can also potentially be deliberately induced to prevent or reverse OA [46,55]. The concept of harnessing the cartilage ECM mechanics to prevent macrophage-mediated initiation of OA has only recently been suggested as a proof-of-concept study that showed that certain stiffnesses could be protective against MF activation in inflammatory OA [45]. Generally, stiffer environments elicit a pro-inflammatory M1 phenotype, while softer environments induce polarization to a pro-remodeling M2 phenotype [53,56]. This relationship between MFs and ECM dynamics could be exploited towards reversing OA or developing novel treatments for MF-mediated OA. More studies are needed to understand the role small changes in the ECM mechanical environment plays in initiation and propagation of OA. Taken together, recent studies illustrate how the ECM can be engineered to enhance cell participation in articular cartilage tissue repair or otherwise support cell delivery [54].

#### 2.2.1. Cartilage ECM as an Engineered Material

The ECM has outsized role in cartilage homeostasis and dysregulation. The specialized ECM found in cartilage is a crucial component of the tissue structure and function, accounting for 90% of the tissue’s dry weight, a higher proportion than what is found in most other tissues, except for tendon and perhaps bone [57]. Chondrocytes account for only 2–5% of total cartilage tissue volume [58]. Due to the unique composition of its ECM, cartilage withstands the high loads experienced during human locomotion [59]. Numerous ECM-mediated environmental factors influence chondrocyte metabolic activities, including composition of the matrix and the secretion of soluble mediators. Growth factors and cytokines are secreted in response to mechanical loading, which is also transmitted to the cells via the matrix [59,60]. Since articular cartilage is avascular, the ECM plays a disproportionately large role in regulating chondrocyte metabolism compared to tissues with higher vascularization.

Cartilage ECM further supports the specialized mechanical properties of the tissues that make articular cartilage engineering a challenge [61]. Articular cartilage has a low coefficient of friction (COF), which is challenging to replicate in engineered constructs. Due to its influence on the COF of engineered cartilage [62], the ECM has emerged as a possible material for lowering the COF of in vitro cartilage constructs. Decellularized ECM extracted from the superficial zone cartilage of juvenile bovine femoral condyles increased the compressive and tensile stiffness of self-assembled articular cartilage constructs prepared from juvenile bovine stifle joints. Importantly, the addition of ECM decreased both the COF and glycosaminoglycan content, and it increased the collagen content of the constructs [63]. This approach demonstrated that the ECM may address the longstanding challenge of mimicking the COF of cartilage. As the COF has been shown to be higher in hydrogel-based biomaterials used to treat articular cartilage defects [64], other materials, such as those fabricated from ECM, may be more appropriate if COF is the main concern. Indeed, ECM scaffolds for cartilage regeneration have generated considerable interest and are reviewed thoroughly elsewhere [3]. More work is needed to understand how to maintain a native cartilage-like COF in vitro and in vivo, particularly over long periods of time. Engineered ECM may be an optimal approach for recreating the appropriate COF.

Human ECM from allogeneic sources [65] or cadaveric donors has also shown promise as an engineered material for cartilage regeneration. An acellular three-dimensional interconnected porous scaffold derived from human cartilage ECM improved cell adhesion, proliferation, and differentiation of bone marrow-derived mesenchymal stem cells (BMSCs) into chondrocyte-like cells over 21 days [66]. These chondrocyte-like BMSCs were transferred onto another cartilage ECM-derived porous scaffold and implanted subcutaneously into nude mice. Over four weeks, these scaffolds produced cartilage-like tissue with high levels of sulfated proteoglycans and collagen type II, illustrating that human ECM can support chondrocyte maturation [66]. Though human ECM is in short supply, small amounts may be sufficient to initiate the proliferation and maturation of chondrocytes needed to trigger endogenous regeneration of cartilage.

Moving forward, cartilage ECM may be purposefully generated from animal or human cells to avoid the need for a donor altogether [67]. A recent study tested decellularized human bone marrow mesenchymal stem cell-derived extracellular matrix (hBMSC-ECM) as a culture substrate for chondrocyte expansion in vitro, as well as a scaffold for chondrocyte-based cartilage repair [68]. Chondrocytes were grown on both tissue culture plastic (TCP) and decellularized hBMSC-ECM. Chondrocytes deposited on the hBMSC-ECM showed significantly increased proliferation rates and effectively maintained the chondrocyte phenotype when compared to the group cultured on TCP. Additionally, chondrocytes in the ECM group had improved chondrogenic differentiation profiles compared to the TCP group controls. To test the in vivo compatibility of the ECM, a three-dimensional chondrocyte impregnated hBMSC-ECM (cell/ECM) culture system was implanted into SCID mice. Fourteen days post-implantation, prominent cartilage formation was observed in the cell/ECM group. These findings suggest that the stem cell-ECM combinatorial approach is a promising avenue for in vitro and eventual in vivo cartilage regeneration.

In addition to serving as the main scaffold material, cartilage ECM can augment other commonly used scaffolds, including polyurethane. A notable advantage of recently developed waterborne polyurethane-ECM (WPU-ECM) scaffolds is that they can be three-dimensionally printed at temperatures suitable for cell survival [69]. These scaffolds successfully achieved hierarchical macro-microporous structures. Following ECM addition, WPU scaffolds had optimal porosity, hydrophilicity, and bioactivity. These scaffolds also enhanced cell distribution, adhesion, and proliferation compared to control WPU-only scaffolds. Most importantly, the WPU–ECM scaffold could facilitate the production of glycosaminoglycan (GAG) and collagen, as well as the upregulation of cartilage-specific genes, which is difficult to achieve in other cartilage tissue engineering platforms. In vivo studies in rabbits showed that WPU-ECM scaffold combined with a microfracture procedure successfully regenerated hyaline cartilage six months after implantation [69]. Overall, although ECM was not the main component of these scaffolds, the combination of ECM with WPU significantly increased scaffold functionality, creating a favorable microenvironment for cell adhesion, proliferation, differentiation, and ECM production. Given the difficulties in sourcing large quantities of ECM, the ability to functionalize readily available scaffold materials, such as polyurethane, with smaller quantities of ECM, while still achieving significant regeneration and repair of the target tissue, is highly desirable.

#### 2.2.2. Cartilage ECM to Modulate Fibrosis

Finally, we examine recent progress in engineering the cartilage ECM to control the progression of fibrosis. OA progression results in increased deposition of fibrotic tissue throughout the joint. As cartilage tissue is primarily ECM, with few chondrocytes, blood vessels, lymph nodes, or nerves, fibrotic tissue overwhelms intrinsic repair responses. The quantity and density of the cartilage ECM further prevents normal healing due to the reduced diffusion of stem cells and nutrients through the tightly packed extracellular and pericellular spaces [70]. In response to the challenges that the ECM poses to healing, studies have attempted to treat fibrosis through the replacement or modification of the cartilage ECM. Drawing inspiration from studies outside of the musculoskeletal system that inhibited fibrotic YAP signaling by using collagenase to soften the ECM in uterine fibroids [71], pro-fibrotic pathways were successfully inhibited using the anti-fibrotic agent nintedanib to protect chondrocytes from fibrosis due to ECM degradation [72]. Other methods for repairing or preventing fibrosis in cartilage have incorporated MSCs, using implanted cells to increase the production of functional ECM and fill in cartilage lesions to replace fibrotic regions [73]. By recalibrating the balance between chondrocyte-generated, functional ECM and fibrotic ECM, such approaches seek to shield the underlying articular tissue from further damage and overloading. Despite their promise, techniques seeking to regenerate the ECM struggle to adequately direct migration of implanted cells, suggesting crucial signals are still missing. Future approaches should engineer the ECM to better enable targeted migration of implanted cells to the desired cartilage areas.

The mere presence of cartilage ECM in engineered constructs promotes further excretion of ECM from chondrocytes. This can be a useful tool in cartilage reparative techniques, as the implanted ECM stimulates the chondrocytes’ reparative response. A survey of various scaffolds for cartilage reconstruction found that growing MSCs in extracted ECM scaffolds resulted in higher chondrocyte proliferation and cartilage ECM production compared to a pure collagen scaffold, and this was conducted without the need for extrinsic growth factors [74]. The chondrogenic effect of the matrix may be due to the natural ECM material’s retention of GAGs, particularly chondroitin sulfate and aggrecan [74]. These proteins have been shown to induce chondrogenesis in in vitro cell populations [75]. As more approaches utilize scaffolds incorporating natural ECM components, improved control over chondrocyte behavior and targeted, chondrogenic regeneration will be achieved. 

Another key aspect of cartilage ECM is the basement membrane, which is responsible for cellular adhesion to the articular interstitial matrix. The basement membrane supports cell signaling, mechanotransduction, and acts as a protective barrier for the cells. Cartilage basement membrane consists primarily of Collagen IV (Col IV), laminin, nidogen, and perlecan. The proportions of these components in cartilage vary due to aging and overall tissue health. Perlecan is of particular interest, as it is a developmental regulator of articular cartilage, binds with a variety of ligands in the ECM and pericellular matrix, and is involved in mechanotransduction and intracellular communication. While the understanding of how perlecan and other basement membrane components contribute to healthy articular cartilage is limited, a recent study found that adequate perlecan in both the basement membrane and pericellular matrix greatly enhanced cartilage repair and structural integrity of the tissue [76]. Perlecan increases around cartilage injury sites, but its role in articular cartilage repair mechanisms remains understudied. More work is needed to understand how this promising ECM component may support articular cartilage engineering.

**Figure 2:**
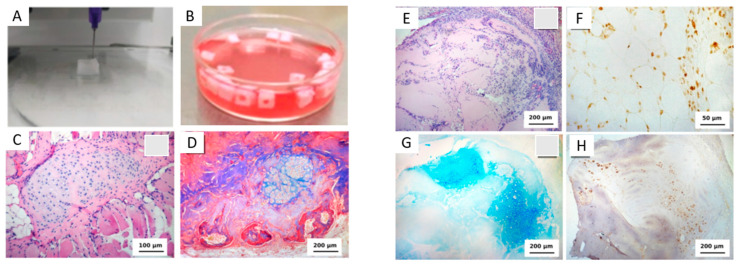
Recent approaches to regenerate articular cartilage have used cartilage ECM and ECM-component ink, in combination with cells. (**A**,**B**) The printing process uses high concentration collage and chondrocyte ink, and the results in scaffolds retain their three-dimensional shape (as shown in (**B**)). (**C**) Hematoxylin and eosin staining of the cartilage tissue that formed after 12 days in culture. Cartilage formation is observed within the striated muscle tissue (20× magnification). (**D**) Mason’s trichrome staining shows bone tissue formation (in the lower section of the image; 10× magnification). (**E**) Five days after implantation, (**H**,**E**) staining shows cellularity within the scaffold (10× magnification). The scaffold in five days after the implantation. (**F**) PCNA-positive nuclei of chondroblasts and proliferating cells in the connective tissue capsule (40× magnification). (**G**) Glycosaminoglycans staining in blue shows cells are producing components of native cartilage matrix (10× magnification). (**H**) Staining for type II collagen (brown) shows additional components of the cartilage matrix are being produced by the metabolically active cells (10× magnification). Taken together, three-dimensional printing of cartilage with custom bioinks is a recent and viable strategy for cartilage tissue engineering studies. Adapted from Beketov et al. (2021) with permission under a Creative Commons CC BY 4 license [78].

Overall, substantial challenges remain in engineering cartilage ECM to prevent or reverse OA and other cartilage diseases. Promising future approaches build upon a strong and expanding foundation of ECM-material combinations that can recreate crucial mechanical and biochemical aspects of healthy and diseased articular cartilage. Unique approaches using bioinks [77,78] (Figure 2) and ECM from organisms outside the mammalian kingdom are also promising [79], adopting research directions that are “outside the box” of traditional tissue engineering strategies. For the first time, emerging techniques, such as three-dimensional printing and electro-writing for scaffold fabrication, can recreate some of the differences observed in natural cartilage layers [77]. An ongoing challenge will be engineering cartilage ECM to possess the unique properties of native tissue, namely, the mechanics and low COF. As new materials emerge and are incorporated into combinatorial scaffold-ECM approaches, musculoskeletal tissue engineering will progress towards functional regeneration of articular cartilage.

### 2.3. Tendon

Selected approaches utilizing cell–ECM–material interactions to regenerate tendon are summarized in Table 3. A tendon is a collagenous musculoskeletal tissue that connects muscle to bone to enable normal movement and daily activity. The tendon is relatively acellular and comprised mainly of Collagen 1 (Col1) that is secreted by tenocytes (tendon cells) during early embryonic and post-natal development. A mature tendon has limited regenerative capacity. Damage to the collagenous tendon ECM is associated with long-term pathology and diminished daily function. Due to its low cellularity, the tendon heals poorly, and this is often accomplished via fibrotic scar formation that prevents normal mechanical function. The precise role of the ECM in tendon development, homeostasis, and disease is multifaceted and remains incompletely understood, but tendon ECM is known to support various mechanical and cellular functions [80]. A critical component of the tendon ECM is its hierarchical organization of Col1 fibers. Disruptions to the structure of these fibers are associated with tendon dysfunction and disease [81]; an important consideration when engineering the tendon ECM is the three-dimensional organization of the matrix components. Non-collagenous components of the tendon ECM must also be considered, as they have numerous functions in aging and disease [82,83].

**Figure 3:**
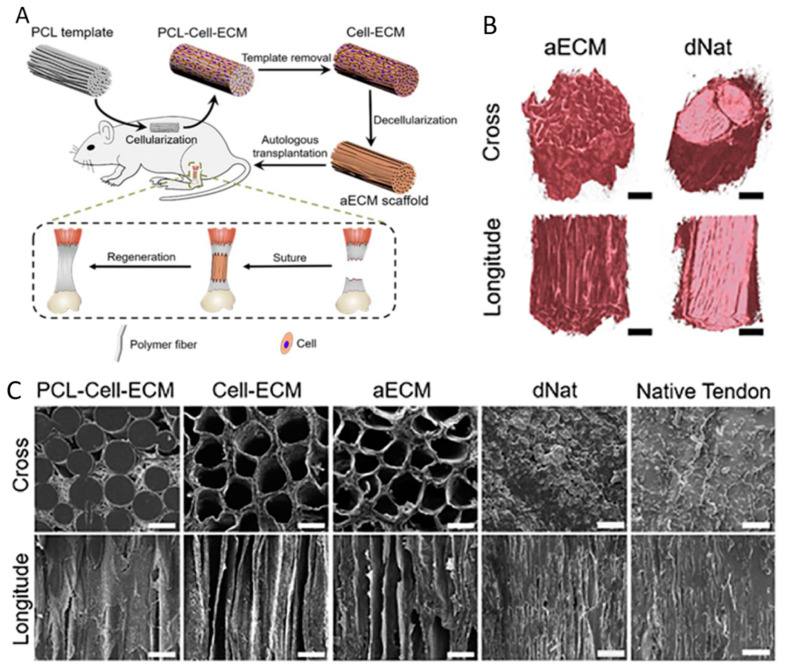
Polycaprolactone (PCL)–cell–ECM scaffolds support tendon regeneration. (**A**) Schematic of the scaffolds and implantation procedure. (**B**) Micro-CT scans of the transverse and longitudinal position in the aECM and dNat scaffolds. (**C**) PCL–cell–ECM, cell–ECM, aECM, decellularized native tendon (dNat) scaffold and native tendon microstructures were visualized using SEM cross- (top panels) and longitude- (bottom panels) sections. The figure was adapted with permission from Li et. al. 2019 [85].

A recent study addressed the need for hierarchical structure in engineered tendon by developing engineered and aligned autologous ECM scaffolds (Figure 3) [84]. Precision-designed templates were subcutaneously implanted in rats to generate decellularized autologous ECM from rats. The resulting scaffolds had highly aligned microchannels, which persisted after the templates and cellular components were removed. Once implanted, the scaffolds promoted rapid cell infiltration, modulated the MF response, supported collagen-rich ECM synthesis, and resulted in physiological tissue remodeling in Achilles tendon defects [84]. Three months post-operatively, the mechanical strength of the tenocyte-populated ‘neo-tendons’ was comparable to that of the pre-injury tendons. This study was one of the first to utilize subcutaneous ECM engineering, and it lays the groundwork for similar approaches in other animals and human patients, where autologous ECM might also be grown subcutaneously.

Overuse injuries are common in tendons. Tendon overuse gradually degrades the ECM and alters its crucial mechanotransductive properties, causing pain and disability [85]. Mechanotransduction is the conversion of mechanical signals to cellular signals; damage to the ECM results in warped signals reaching the cells, in turn impacting the cellular response to the loads caused by daily movement. Continuous microdamage accumulation in the tendon ECM, combined with the tissue’s poor regenerative capabilities, contributes to tendinopathy. Engineered tendon ECM must account for the wide variety of mechanical stimuli different tendons experience (for example, force-transferring Achilles tendons versus limb-positioning digital flexor and extensor tendons) and their reaction to these loading profiles [86,87]. Tendon tissue engineering is further complicated by the poor understanding of how distinct tendons may follow different developmental pathways, and they may vary in certain cellular processes, such as ECM homeostasis and remodeling [88]. While cell–EMC interactions are generally mediated by structures including cilia, integrins, connexins, and the cytoskeleton to support homeostasis, adaptation, or repair, it is possible, and perhaps likely, that these structures are not consistent across all tendon types. Attempts to engineer tendon ECM for use in regenerative studies must consider the unique functions of the target tendons. Such approaches have yet to be explored, but they are a promising future direction for tendon tissue engineering. 

#### 2.3.1. Engineered Tendon ECM to Understand Development and Disease

Mechanical force regulates tendon ECM organization, as well as tendon growth factor signaling through the transforming growth factor beta (TGFβ) family [89], indicating that tailoring the ECM to modulate the mechanical signals received by tendon cells is a crucial consideration of tendon tissue engineering. Work in zebrafish showed that, in addition to being transmitted via the ECM, mechanical forces can change the composition of the tendon ECM [89]. The feedback between tendon cells and ECM composition in response to mechanical loading supports the need to utilize ECM in tendon tissue engineering as a means of delivering desired signals to the cells. Multiscale mechanotransduction via the ECM has been proposed as a central focus of future tendon therapeutics [88]. 

An engineered tendon ECM can also be used to direct stem cell differentiation towards the tendon phenotype. The extracellular compartment is responsible for tendon morphogenesis during early development, in part due to the spatial cues it provides [90]. ECM elasticity [91] and proteoglycan content [92,93] have also been extensively implicated in tendon development and disease, but have yet to be considered as tunable and targetable components of engineered tendon ECM. As the tendon ECM is highly specialized [94], tendon tissue engineering may benefit by exploiting this specialization and incorporating specific ECM components in both in vitro and in vivo work. Future work that designs an ECM consisting of selected ECM components and other molecules found to be critical to tendon differentiation, such as cadherins and connexins [95], can elucidate the unknown cellular and mechanical processes that underlie tendon disease. 

Beyond its mechanical properties, tendon ECM has been engineered to support tissue function via enhancement of its structural and compositional functions. Solubilized tendon ECM was recently used to induce tenogenesis (differentiation towards tendon) in human adipose-derived stem cells [96]. Extracted tendon ECM yielded consistent protein compositions that enhanced tenogenic gene expression in the cells via pathways related to ECM-associated processes. These findings support the continued use of solubilized ECM-based materials and approaches for enhancing musculoskeletal differentiation from stem cells. Such approaches may prove especially useful for induced pluripotent stem cells (iPSCs), in which consistent control of differentiation has proved challenging. ECM derived from specific tissues may provide cues that are otherwise difficult to incorporate within in vitro studies. In addition to providing specific matrix components, engineered ECM may also supply degradation cues, in response to which the cells can initiate remodeling. Such an approach may approximate the degradation–regeneration cycle seen with exercise [97]. Mimicking these cues in vitro via targeted ECM modifications can establish what amount of exercise is protective versus damaging to tendons in various stages of development or healing.

**Table 2:** Recent and noteworthy studies focused on cell–ECM–material interactions in cartilage.

Tissue	Model	Material	Main Findings
Cartilage	Rat bone marrow-derived mesenchymal stem cells (rBMSCs) cultured with cryo-ground decellularized cartilage ECM.	Cryo-ground decellularized cartilage ECM.	Chemically decellularized cartilage (DCC) particles significantly outperformed TGF-β in chondroinduction of the rBMSCs. Collagen II gene expression was more than an order of magnitude greater compared to controls [74].
	Porcine methacryl-modified solubilized and devitalized cartilage (MeSDVC) hydrogels.	Cryo-ground decellularized cartilage ECM methacrylated with glycidyl methacrylate (GM) and methacrylic anhydride (MA).	Methacrylation of the ECM increased printability of the MeSDVC hydrogels by creating paste-like consistency. Hydrogel stiffness increased to physiologically useful ranges [98].
	BMSCs grown in dual-stage crosslinked hyaluronic acid-based bioink that was covalently linked to transforming growth factor-beta 1 (TGF-β1).	Hyaluronic acid (HA) bioink with covalently bonded TGF-β1.	Tethered TGF-β1 maintained functionality post three-dimensional printing and generated high quality cartilaginous tissues without exogenous growth factors [99].
	BMSCs grown in porcine photocrosslinkable methacrylated cartilage ECM-based hydrogel bioink (cECM-MA).	Decellularized MA- methacrylated cartilage ECM bioink.	BMSCs were viable post-printing and underwent chondrogenesis in vitro, generating tissue rich in sulphated glycosaminoglycans and collagens [100].
	Rat chondrocytes grown in genipin-crosslinked gelatin scaffolds with varying porosity.	Genipin-crosslinked gelatin scaffolds.	Chondrocytes proliferated and readily generated ECM with pore sizes of 250 and 500 μm [101].
	hMSCs grown in tunicate exoskeleton-derived dECM.	Tunicate dECM.	Tunicate ECM was decellularized while retaining the honeycombed-shaped microstructure that improved metabolic activity, cell proliferation, and chondrogenic differentiation in hMSCs [79].
	Rat chondrocytes grown in high concentration collagen bioprinted hydrogel scaffolds.	An amount of 4% collagen hydrogel bioink.	Subcutaneous implantation of the bioprinted scaffold resulted in cartilage-like tissue formation in rats as early as one week post implantation [78].
	BMSCs grown in polyethylene glycol diacrylate (PEGDA) and ECM electro-written hydrogel.	High porosity PEDGA and porcine-derived ECM electro-written scaffold.	Electro-written PEDGA and ECM scaffold induced chondrogenesis and had anti-inflammatory effects [79].
	Adipose-derived stem cells (ADSCs) grown in cartilage dECM and waterborne polyurethane (WPU) scaffolds, using low-temperature deposition manufacturing (LDM).	Cartilage dECM and WPU.	Hierarchical macro-microporous dECM- WPU scaffolds regenerated hyaline cartilage in a rabbit articular cartilage microfracture model [69].
	Mouse chondrocytes in human bone marrow-derived MSC-ECM (hBMSC).	hBMSC-ECM.	In vivo subcutaneous implantation of hBMSC-ECM scaffold in mice improved chondrocyte proliferation and development of a bioactive matrix [68].
	Decellularized allogeneic hyaline cartilage graft (dLhCG) for porcine knee repair.	Decellularized pure hyaline-like cartilaginous ECM.	dLhCG resulted in superior efficacy in articular cartilage repair, surpassing living autologous chondrocyte-based cartilaginous engraftment repair methods [65].
	Self-assembled articular cartilage constructs grown in bovine femoral condyle superficial zone cartilage ECM.	Bovine femoral condyle superficial zone cartilage ECM.	Extracted cartilage ECM reduced friction coefficients of the self-assembled articular cartilage constructs [63].

#### 2.3.2. Engineered Tendon ECM as a Repair Material

As tendon ECM provides essentially all the mechanical functions of tendon tissue, replicating its structure and function is of particular importance to regenerative and tissue engineered approaches for treating tendon injuries. The acellular matrix has been explored as a means of augmenting rotator cuff repairs, where disruption to the gradated tendon-mineralized fibrocartilage-bone matrix is associated with complex recoveries and long-term loss of function. Human acellular dermal matrix (ADM) used as a patch was shown to be a viable option based on results in canine large rotator cuff defect models [24]. More recently, bovine decellularized tendon matrix (DTM) used in a rabbit model of Achilles tendon injury successfully prevented post-surgical adhesion, a common complication following surgical tendon repairs [98]. DTM acts as an anti-adhesion membrane, with the added benefit of being completely degraded after 12 weeks of subcutaneous implantation. This same study pioneered the use of tandem mass tag (TMT) labeling proteomics to analyze the protein compositions of native tendon, acellular tendon, and DTM, so as to quantitatively show the significant bioactivity and regenerative potential of the bovine DTM, and this is likely for other DTM-type materials. Recent advances in decellularization techniques, combined with micro-sectioning to preserve tissue viability and bioactivity with reduced reliance on chemical saturation, are expanding the pool of viable ECM sources. The emerging concept of using engineered ECM materials not only as a component of scaffolds, but also as a post-surgical treatment to augment existing repair techniques, will expand the potential applications of engineered ECM in the coming decades. 

Another promising approach in tendon repair is the use of ECM to address post-operative fibrosis. Tendon surgery can lead to fibrosis of the affected or implanted tissue due to the graft adhering to surrounding native tissues. This adherence is the most common post-surgery complication in tendon repair [99], but decellularized ECM shows promise for preventing this outcome. A recent clinical trial showed that freeze-dried amniotic membrane could prevent adhesion following surgical repair of flexor tendons injured in zone II [99]. Adhesion was significantly lower in patients with the amnion membrane wrapped around the ends of the tendon compared to control patients who received implants made of poly-DL-lactic acid (PDLLA). Immune responses were also minimal in the amnion group, suggesting that this method may be used to prevent post-surgical tendon adherence in the future, as well as in larger tendons than the flexors of the hands. Another recent clinical trial also successfully used ADM during hand flexor reparative surgery as a tendon scaffold and tendon adhesion preventative [100]. Twelve months post-operatively, patients treated with the ADM had significantly reduced adhesion and increased tendon functionality compared to controls treated without the implant. A third trial using ADM found improved range of motion of the proximal and distal interphalangeal joints six months post operatively versus control groups treated conventionally [101]. Taken together, these and other studies show that matrix implants can aid in preventing adhesion and halting further tissue and joint destruction following invasive reparative surgeries. More work is needed to characterize the cellular interactions and behaviors that these implants facilitate within the tendons. Once the cellular pathways are better understood, new matrix-based materials may be developed that stimulate desired cellular behaviors and suppress unwanted outcomes, such as activation of the cellular pathways leading to fibrosis and adhesion.

**Table 3:** Recent and noteworthy studies focused on cell–ECM–material interactions in tendon.

Tissue	Model	Material	Main Findings
Tendon	Acellular dermal matrix (ADM) tendon scaffold affixed to hand flexor tendon post-operation.	Decellularized dermal ECM.	Addition of ADM post operation reduced tendon adhesion and improved long term functionality of the flexor tendon [100].
	Decellularized bovine tendon ECM used as an anti-adhesion membrane.	Decellularized tendon matrix (DTM).	DTM improved tendon repair in rabbits by reducing adhesion and cellular proliferation, as well as improving healed tendon quality [98].
	Human adipose-derived stem cells (hASCs) grown in urea-extracted bovine decellularized tendon matrix (DTM).	Urea-extracted decellularized tendon matrix (DTM).	Urea-extracted DTM increased hASC proliferation and tenogenic differentiation, and it also induced unique tenogenic gene expression profiles [96].
	Rat tendon self-repair with implanted decellularized autologous extracellular matrix (aECM) scaffolds with highly aligned microchannels.	aECM scaffolds with aligned microchannels created through poly (ε-caprolactone) (PCL) microfiber bundle templates.	Subcutaneously implanted aECM scaffolds with aligned microchannels increased cellular infiltration and proliferation in the damaged tendon, resulting in improved restoration of rat tendon post-injury [84].
	Human acellular dermal matrix graft for canine tendon repair.	Decellularized dermal ECM.	Within 12 weeks of implantation, the graft restored tendon functionality and mimicked autologous tendon both histologically and mechanically [24].

#### 2.3.3. Engineering Cell Interactions with the Non-Collagenous Components of the Tendon ECM

It is worthwhile to consider the interactions between the cells and the non-collagenous components of tendons. While collagens and their hierarchical structural formations make up 70 to 90% of the total tissue volume in tendon, its less abundant components also provide crucial functions. These include proteoglycans, glycosaminoglycans, and non-collagenous proteins that are incompletely understood and poorly characterized, particularly with respect to their roles in mechanotransduction and their contributions to the mechanical properties of tendon [88,102]. These non-collagenous components are important for mediating cellular interactions with the matrix. The pericellular matrix directly surrounding tendon cells is comprised of mostly non-collagenous proteins that transfer mechanical stimuli to the cells in order to regulate mechanosensitive expression of genes [102]. To date, no approaches have directly engineered the makeup of the tendon ECM to quantify how specific cellular interactions with specific non-collagenous proteins affect tendon development, homeostasis, injury, or repair. There is significant potential for future approaches to manipulate the non-collagenous components of tendon ECM with the goal of directing specific cellular responses for tendon regeneration.

Engineered cell–ECM–material interactions may also benefit tendon wound repair. The tendon has limited healing capacity, but prior work has shown that biglycan and fibromodulin, two non-collagenous components of the tendon ECM, can enhance the wound healing capacity of tendon progenitor cells [103]. Recent work has shown that interactions with both the collagenous and non-collagenous components of the tendon ECM supports proliferation and wound healing properties of tendon stem/progenitor cells [104], while a new study also demonstrated the potential of the hippo-pathway downstream effector, yes-associated protein (Yap), as a signaling target using exosomes for targeted wound repair [105]. Notably, hippo and Yap are known to be mediated by mechanical and biochemical signals from the ECM. The results of this study indicate that exosomes stimulating these pathways could be a way to bypass the need for a healthy ECM to drive wound healing, in the case that there is extensive damage to the tendon ECM following injury or disease. More work must be performed regarding the use of exosomes in moderating and enhancing certain cell-ECM interactions.

Ultimately, though the tendon may be the least explored tissue in the context of engineered ECM approaches for regeneration, we see rapid advancements in the use of natural and engineered ECM and ECM-composite materials for uses in tendon tissue engineering. As ongoing research elucidates the impact of the ECM in tendon development, maintenance, and injury, engineered ECM-material approaches will provide useful interventions for improved tendon healing and regeneration. Tendon ECM is responsible for many of the tissues’ mechanical and biochemical properties, and expanding the capabilities of engineered ECM to drive cell behavior is a critical and ongoing area of investigation.

### 2.4. Bone

Selected approaches utilizing cell–ECM–material interactions to regenerate bone are summarized in Table 4. Skeletal tissues serve important functions related to overall health, most notably providing the structure and protection necessary for the internal organs to perform their vital functions. Bone ECM has additional roles as a reservoir of calcium and other inorganic ions [106]. The cells housed within the bone matrix are major and active regulators of calcium homeostasis, and they secrete important hormones in response to cues from the ECM [106]. Bone is a regenerative tissue that is continuously remodeled throughout the lifespan by the osteoblasts on the bone surface that deposit new matrices, and the osteoclasts resorb the matrices. Osteocytes, the terminally differentiated cells embedded within the bone, maintain bone homeostasis and orchestrate many endocrine and paracrine functions [107]. Much of the interest in the bone matrix is driven by the search for improved treatments for osteoporosis, a bone-mass loss and demineralization disease that impacts hundreds of millions of patients globally [108]. Osteoporosis, more common in women over 50 due to menopause, reducing bone density, leads to decreased bone mass that can result in frequent fractures, particularly of the spine, arms, and hips [109]. Understanding how the bone ECM contributes to disease prevention, progression, and potential prognosis [110] is an urgent clinical need. To this end, several approaches have used bone-derived ECM scaffolds to understand how the bone matrix impacts cell behavior and bone regeneration.

#### 2.4.1. Engineered Bone ECM-Mimicking Scaffolds

The well-defined composition and structure of bone ECM facilitates biofabrication of bone ECM scaffolds for uses in tissue engineering. Mimicking the porosity, permeability, and tortuosity of bone ECM is relatively straightforward and may enhance cell migration, as show in a recent computational study [111]. Another study integrated distinct biofabrication strategies to develop a multiscale porous scaffold that was both mechanically functional at the time of implantation, and that facilitated rapid vascularization, while providing stem cells with appropriate cues to enhance differentiation into osteoblasts [112]. A polycaprolactone (PCL) scaffold was integrated with decellularized bone ECM, producing osteoinductive filaments for three-dimensional printing. The addition of bone ECM to the PCL not only increased the mechanical properties of the resulting scaffold, but it also improved cellular attachment and enhanced osteogenesis of mouse mesenchymal stem cells (MSCs). The use of three-dimensional printing, particularly for bone ECM with its stable, interconnected, and somewhat predictable structure, will likely feature prominently in ongoing studies seeking to enhance cellular bone deposition.

Oriented ECM scaffolds have also been fabricated to imitate the material and structural properties of the natural bone growth plate, utilizing bone marrow stromal cells (BMSCs) to prevent bone bridge formation and growth plate injuries [113]. Growth plate injuries can disrupt normal bone development and lead to irregularities in bone length and shape after healing [114]. Limited treatments exist for growth plate injuries, especially if intervention is not rapid [115,116]. A recent study induced a growth plate injury in rabbits. Animals were treated with engineered, oriented ECM scaffolds and autogenous BMSCs, ECM scaffolds only, or injured but not treated with a scaffold or cells. At 16 weeks, the tibial defects (simulated growth plate injuries) treated with the ECM scaffolds and cells were filled with a neogenetic growth plate. Rabbits treated with only the ECM scaffolds still had physical defects, but the growth plate displayed some closure, with bone trabeculae and fibrous tissue growth. Controls showed some closure of the growth plate. Overall, BMSCs successfully adhered to and distributed within in the oriented scaffold in the group treated with both the ECM scaffold and the cells [117]. Radiological testing further showed that the scaffold and cell treatment decreased angular deformities and length discrepancy of the tibia when compared to other groups. The addition of BMSCs within the ECM scaffolds also promoted the regeneration of neogenetic chondrocytes during the repair of the injured growth plates and prevented the formation of bone bridges. Although total prevention of angular deformities and length discrepancies was not achieved, ECM-derived growth plate scaffolds combined with BMSCs have exciting potential applications in growth plate repair. 

ECM from non-musculoskeletal tissues has also been successfully used to induce in situ bone regeneration. Porous polycaprolactone (PCL)/decellularized small interesting submucosa (SIS) scaffolds were fabricated using cryogenic free-form extrusion, followed by surface modification with aptamer and PlGF-21_23–144_peptide-fused bone morphogenetic protein 2 (pBMP2) [118]. Rats were used to model a critically sized calvarial defects, into which a scaffold was implanted. At four- and eight-weeks post-operatively, defects implanted with the PCL/SIS-BMP2-Apt and PCL/SIS-pBMP2-APT scaffolds had substantial mineralized tissue, which was not seen in defects implanted with PCL/SIS and PCL/SIS-Apt groups. Micro-CT showed that the bone volume/tissue volume percentage (BV/TV%) and bone mineral density (BMD) values for defects implanted with PCL/SIS-Apt were significantly higher than those in the control group, suggesting that scaffolds with aptamers stimulated bone regeneration in vivo [119]. At eight weeks post-operatively, the bones in the injury sites with the PCL/SIS-pBMP2-Apt scaffold had completely bridged the defect. Bone formation was not seen in the PCL/SIS group, where only connective tissue formed. Rats implanted with the PCL/SIS-pBMP2-Apt scaffolds also showed evidence of angiogenesis. The combined use of BMP2 and Apt-19 (Aptamer 19) within a biomimetic PCL/SIS scaffold has promising applications in bone regeneration, despite the ECM source (the SIS) being outside the musculoskeletal system. Using SIS in musculoskeletal regenerations may be advantageous compared to other types of ECM, as large quantities of SIS are readily available.

Another promising scaffold for bone regeneration was recently developed using polylactic glycolic acid, bone ECM, and magnesium hydroxide. Magnesium is an emerging scaffold material that resists the loading from human activities and has potential uses as an orthopedic implant due to its porosity and robustness [120]. To mediate the negative effects associated with PLGA (i.e., low mechanical properties and acidic byproducts), a porous PLGA (P) scaffold was created and combined with magnesium hydroxide, (MH, M) bone-extracellular matrix (bECM, E), and polydeoxyribonucleotide (PDRN, P) to improve anti-inflammatory ability, osteoconductivity, and introduce pro-osteogenic and pro-angiogenic effects, as well as osteoclast inhibition [121]. PLGA (control), PME, and PMEP groups were assessed for biocompatibility and cellular activity. PME and PMEP groups had significantly increased biocompatibility compared to the PLGA group, as both scaffolds increased the population of calcein AM positive human bone-marrow mesenchymal stem cells (hBMSCs), i.e., live cells, at one, three, and seven days post-implantation [117]. The angiogenic properties of the PDRN were confirmed using human umbilical vein endothelial cells (HUVECs) treated with PDRN, since PDRN treatment formed a significant number of branch points and longer vessel lengths in the HUVECs. Quantitative real-time PCR (qRT-PCR) determined the that the PME scaffold reduced inflammation markers in the 3D hBMSC scaffolds at seven and twenty-one days, since expression of interleukin-6 (IL-6) and interleukin 1β (IL-1β) were lower compared to the PLGA scaffolds. The PMEP scaffold further reduced inflammatory markers when compared to PME scaffolds. Osteogenic capacity was also highest in the PMEP scaffolds. These findings show that mMH has the potential to both enhance the mechanical properties and neutralize acidification of PLGA scaffolds, with widespread implications for bone tissue engineering, as PLGA has potential to be an excellent material for bone regeneration if its mechanical properties and acidification are improved. bECM improved osteogenesis by effectively providing natural calcium and phosphate. The vascularization driven by the PDRN underscores the potential applications of these engineered, ECM–cell–material-based scaffolds. 

#### 2.4.2. Bone Scaffold Functionalization with ECM

In addition to serving as a scaffold material, ECM can functionalize existing scaffolds to enhance osteogenic potential for bone regeneration applications. As a recent example, ECM was functionalized onto the surface of multi-channel biphasic calcium phosphate granules (MCG) to increase osteogenesis and regeneration. The collagen, sulfated glycosaminoglycan, and trace amounts of growth factors, such as BMP2, vascular endothelial growth factor (VEGF), transforming growth factor TGFβ, and fibroblast growth factor (FGF), were found in the ECM supported proliferation of MC3T3-E1 cells over seven days [118]. Protein adsorption and osteogenic properties were improved on ECM functionalized MCG scaffolds compared to controls, and ECM functionalized scaffolds enhanced bone regeneration in a rabbit model of a femoral head defect. While this particular study functionalized MCG scaffolds only, ECM can be formatted into powders, gels, sponges, bioinks, and other formats to improve existing biomaterial scaffold systems used for musculoskeletal regeneration. 

**Figure 4:**
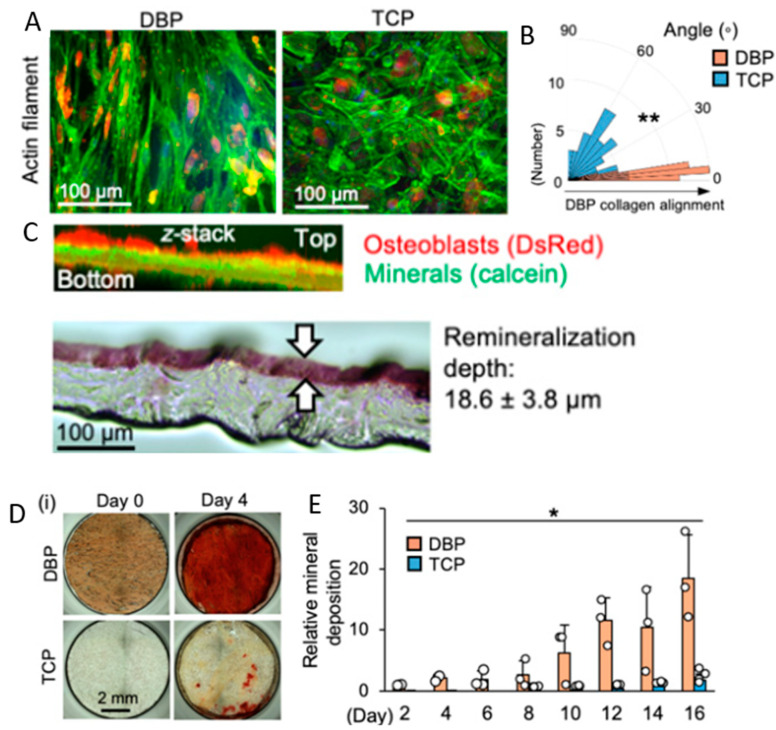
Osteoblasts grown on demineralized bone paper (DBP) display enhanced regenerative capacities compared to osteoblasts grown on tissue culture polystyrene (TCP). (**A**) Morphology of OBs grown on vertically sectioned DBP and TCP for one week. (**B**) Immunofluorescent staining of actin filaments. (**C**) Circular histogram of cell alignment angles (*n* = 100). (**C**) Top: *z*-staked cross-sectional image of DBP will osteoblasts growing. Bottom: Cross section of 100-μm-thick DBP stained with alizarin red after three-week culture of osteoblasts (*n* = 3). (**D**) Mineral deposition by osteoblasts on DBP and TCP, showing alizarin red mineral stain on days zero and four. (**E**) Time-course measurement of mineral deposition by osteoblasts for 16 days (*n* = 3). * indicates *p*-value < 0.05; ** indicates *p*-value < 0.01. Figure adapted with permission under a Creative Commons Open Access License from Park et al., 2021 [120].

In a unique formatting approach, bone ECM was configured into demineralized bone paper (DBP) as a material to direct osteoblasts to deposit structural mineralized bone tissue [119]. DBP effectively stimulated the trabecular osteoid, directed rapid, and structural mineralization by osteoblasts, and it contained the microenvironment necessary to support bone remodeling. Notably, this study focused on the trabecular bone niche, rather than the cortical bone niche explored in many other approaches, an important detail in the context of diseases, such as osteoporosis that severely impact the trabecular bone of the vertebrae [108]. Compared to control cells cultured in TCP, which is commonly used in osteogenic in vitro experiments, cells grown in the DBP displayed significantly higher mineralization, collagen alignment, and elongated morphology that was also aligned with the underlying lamellar structure of the demineralized bone [119] (Figure 4). As one of the first studies to create a bone organoid for understanding the mineralization of trabecular bone, this work lays the foundation for future inquiries using a new generation of in vitro bone models.

Other notable in vitro bone models work towards elucidating the mechanisms necessary for proper bone–ECM interactions (alignment and osteogenic differentiation). It was recently established that many ECM-associated proteins contain thrombospondin type 1 repeat (TSR) motifs that play critical roles in cellular processes, such as cell–cell attachment, extracellular matrix (ECM) remodeling, cell proliferation, and apoptosis [120]. TSRs are modified with O-linked fucose that is added in the endoplasmic reticulum to folded TSRs by the enzyme protein O-fucosyltransferase-2 (POFUT2). POFUT2 likely promotes efficient trafficking of substrates. Mouse embryos that lack POFUT2 die early in development, emphasizing the importance of the modification for TSR-protein function. Using Prrx1-Cre recombinase, a recent study investigated the impact of POFUT2 knockout on the secretion of POFUT2 substrates and on ECM properties in vivo. Loss of POFUT2 in the limb mesenchyme caused significant shortening of the limbs, long bones, and tendons, and it stiffened the joints, resembling the deformities seen in musculoskeletal dysplasias in humans and mice with ADAMTS or ADAMTSL mutations [120]. In the mouse model, limb shortening was evident by day 14.5, and loss of O-fucosylation led to an accumulation of fibrillin 2 (FBN2), decreased BMP and IHH signaling, and increased TGF-β signaling. The hypertrophic zone decreased in size, with lower levels of collagen X (ColX). Surprisingly, there were minimal effects of the POFUT2 knockout on secretion of two POFUT2 substrates, CCN2 or ADAMTS17, in the developing bone. Conversely, CCN2, ADAMTS6, and ADAMTS10 (POFUT2 substrates important for bone development) had decreased secretion from POFUT2-null HEK293T cells in vitro. Overall, this study suggests that there are cell-type specific responses to the POFUT2 mutation, and O-fucose modification on TSRs may stretch further than the promotion-efficient trafficking of POFUT2 substrates, with the potential to influence their function in the extracellular environment [120]. Further studies that characterize the mechanisms behind O-fucose modification on TSRs may provide valuable insight to how ECM-associated protein modifications affect bone tissue regeneration. 

### 2.5. Tuning ECM Mechanical Signals for Bone Tissue Engineering

While this review has already discussed the widely accepted premise that ECM stiffness is important in the regulation of stem cell differentiation, how cells sense stiffness cues and adapt their metabolic activities remains less understood. Recent research explores the role of mitochondrial phosphoenolpyruvate carboxykinase (PCK2) in enhancing osteogenesis in three-dimensional ECM via glycolysis in the context of stem cell osteogenesis [121]. The three-dimensional trabeculae network of normal and osteoporotic bone with different microstructure and stiffness were mimicked via encapsulation of bone marrow derived mesenchymal stem cells (BMMSCs) in methacrylate gelatin (GelMA) hydrogels. The osteoporotic mice models were established by ovariectomy and compared with sham controls, where three-dimensional BMMSCs/GelMA complexes were inserted into surgically induced femoral defects. PCK2 promoted osteogenesis in three-dimensional ECM with tunable stiffness in vitro and in vivo [121]. PCK2 enhanced the rate-limiting metabolic enzyme pallet isoform phosphofructokinase (PFKP) in three-dimensional ECM, and it further activated the AKT/extracellular signal-regulated kinase 1/2 (ERK1/2) cascade, which directly regulates osteogenic differentiation of MSCs. The newfound complexity of the crosstalk between cell mechanics and metabolism may inform ongoing approaches seeking to harness kinase cascades for bone regeneration. Elucidating such mechanisms provides a unique direction for ECM-mediated bone tissue regeneration, as well as new therapeutic strategies for osteoporosis.

A similar study used a mouse model of trauma-induced heterotopic ossification (HO) to examine how cell-extrinsic forces impact mesenchymal progenitor cell (MPC) fate. Mice were given a partial thickness scald burn injury to produce a burn/tenotomy HO model. Post injury, mice had their hind limbs immobilized. Single-cell (sc) RNA sequencing of the injury site revealed an early increase in MPC genes associated with pathways of cell adhesion and ECM-receptor interactions, as well as MPC trajectories to cartilage and bone [122]. Interestingly, active mechanotransduction was observed after the injury, with increased focal adhesion kinase signaling and nuclear translocation of transcriptional coactivator TAZ. TAZ inhibition mitigates HO, but joint immobilization was shown to decrease mechanotransductive signaling, which completely inhibited HO [122]. As HO is a debilitating complication following both soft and hard musculoskeletal tissue injury, there is significant interest in inhibiting HO without also inducing permanent bone loss or adipogenesis within musculoskeletal tissues. This study further identified decreases in collagen alignment and increases in adipogenesis as a result of immobilization, highlighting the need for a better understanding of the delicate balance between HO and adipogenesis following bone injury. Overall, this study suggests that joint immobilization after injury causes decreased ECM alignment, altered MPC mechanotransduction, and changes in genomic architecture, favoring adipogenesis over osteogenesis. Understanding the importance of mobilization for correct fiber alignment in the ECM is important for both bone tissue regeneration and the long-term management of other musculoskeletal tissues injuries. 

Faulty skeletal muscle repair can also result in HO. A recent study used fibrodysplasia ossificans progressiva (FOP), a disease of progressive HO caused by an activin receptor type-1 (ACVR1) mutation, to elucidate how ACVR1 affects skeletal muscle repair [123]. Primary FOP human muscle stem cells (Hu-MuSCs) isolated from cadaveric skeletal muscle had increased ECM marker expression, as well as skeletal muscle-specific impaired engraftment and regeneration ability. Human iPSC-derived muscle stem/progenitor cells (iMPCs) single-cell transcriptome analyses from FOP showed uncharacteristically increased ECM and osteogenic marker expression compared to control iMPCs [124]. These findings suggest that ACVR1 activation in iMPCs or Hu-MuSCs may contribute to HO by changing the local tissue environment, and blocking this activation can prevent HO in ECM and non-ECM based regenerative approaches. Alternatively, genetically modified cells with dysfunctional ACVR1 could be useful starting platforms for growing scaled-up muscle tissues in vitro that can be subsequently implanted into VML defects.

Bone ECM stiffness has also been implicated in pathologies of the spine. Intervertebral disc degeneration (IDD) is thought to be initiated by the mechanical stimulation provided by the ECM stiffness. ECM defines the mechanical microenvironment of the nucleus pulposus (NP). The mechanosensitive ion channel Piezo1 mediates mechanical transduction within the disc, and a recent study explored the function of Piezo1 in human NP cells subjected stiffness levels comparable to those found in the ECM. The expression of Piezo1 and the ECM elasticity modulus increased in degenerative NP tissues, and stiff ECM activated the Piezo1 channel and increased intracellular Ca^2+^ levels [124]. Increased intracellular reactive oxygen species (ROS) levels and factors that contribute to endoplasmic reticulum (ER) stress were also observed as a result of Piezo1 activation. Additionally, oxidative stress-induced senescence and apoptosis in human NP cells were correlated with stiffer ECM [125]. By inhibiting Piezo1, senescence and apoptosis were alleviated in the stiff ECM environment. Correlatively, Piezo1 silencing in vivo improved the condition of IDD and decreased the elastic modulus of rat NP tissues. Manipulating Piezo1 may be beneficial in mediating the effects of stiffening ECM that could occur with ECM mediated tissue regeneration techniques.

Moving forward, as the use of scaffolds containing cell-derived ECM continues to be a viable strategy for mediating bone regeneration, a mechanistic understanding of the benefits of ECM compared to other scaffolding techniques is needed. A recent study used ECM-loaded three-dimensional printed gelatin (Gel), sodium alginate (SA), and 58s bioglass (58sBG) gels seeded with either rat aortic endothelial cells (RAOECs) or rat bone mesenchymal stem cells (RBMSCs) were used to explore the mechanisms underlying the advantages of ECM-containing scaffolds. In vitro osteogenic differentiation results showed that scaffolds coated with ECM significantly increased the expression of osteogenic and angiogenic genes compared to uncoated control scaffolds, likely due to the presence of native ECM proteins [125]. In vivo experimentation showed that ECM-loaded scaffolds implanted into mandibular defects effectively promoted bone healing compared to non-ECM scaffolds. Both RAOECs–ECM and RBMSCs–ECM scaffolds greatly enhanced bone formation as a result of multiple factors, namely, the increased expression of RUNX2, OCN, BMP2, CD31, and VEGF [125]. More studies are needed that exploit the ECM specifically to enhance expression of these factors in an osteogenic manner, given they may be the mechanism behind the improved cellular outcomes seen with ECM scaffolds. 

Looking towards the future, with large proportions of the population aging in the United States, China, Japan, and globally, the clinical need for regenerative bone therapies will continue to grow exponentially. We have highlighted unique strategies for exploiting the existing cellular ability to remineralize and regenerate bone, without the need for invasive surgical grafts or drug combinations with side effects that reduce the patients’ quality of life. As research continues to elucidate the mechanisms by which bone ECM supports and regulates bone function, paracrine and endocrine signaling, and homeostasis, engineered ECM approaches will provide novel and exciting platforms for regenerative bone therapies. 

**Table 4:** Recent and noteworthy studies focused on cell–ECM–material interactions in bone.

Tissue	Model	Material	Main Findings
Bone	Polycaprolactone (PCL) scaffold integrated with decellularized bone ECM seeded with mouse mesenchymal stem cells (MSCs).	Polycaprolactone (PCL) scaffold integrated with decellularized bone ECM.	The addition of bone ECM to the PCL increased the mechanical properties of the resulting scaffold, increased cellular attachment, and enhanced osteogenesis of mouse mesenchymal stem cells (MSCs) [112].
	Growth plate injury was induced in rabbits and treated with engineered oriented ECM scaffolds and autogenous BMSCs, ECM scaffolds only, or injured but not treated with a scaffold or cells.	Engineered oriented ECM scaffolds.	BMSCs successfully adhered to and distributed within the oriented scaffold in the group treated with both the ECM scaffold and the cells. The ECM scaffold and BMSCs generated functional tissue-engineered cartilage superior to the other groups, and the scaffold and cell treatment decreased angular deformities and length discrepancy of the tibia when compared to other groups. Addition of BMSCs within the ECM scaffolds promoted regeneration of neogenetic chondrocytes during the repair of the injured growth plates and prevented the formation of bone bridges [113].
	Critical-sized calvarial defect in a rat model; porous polycaprolactone (PCL)/decellularized small interesting submucosa (SIS) scaffolds injected into defect. Scaffolds were fabricated using cryogenic free-form extrusion and surface modification with aptamer and PlGF-2123-144peptide-fused bone morphogenetic protein 2 (pBMP2).	Porous polycaprolactone (PCL)/decellularized small interesting submucosa (SIS) scaffolds.	Four- and eight-weeks post-op, defects implanted with the PCL/SIS-BMP2-Apt and PCL /SIS-pBMP2-APT scaffolds had substantial mineralized tissue not seen in defects implanted with PCL/SIS and PCL/SIS-Apt groups. Significantly higher bone volume/tissue volume percentage and bone mineral density for defects implanted with PCL/SIS-Apt compared to controls. Eight weeks post-op, the bones in the injury site with PCL/SIS-pBMP2-Apt scaffold had completely bridged the defect, and angiogenesis occurred in rats implanted with the PCL/SIS-pBMP2-Apt scaffolds [116].
	Porous PLGA (P) scaffold combined with magnesium hydroxide (MH, M), bone-extracellular matrix (bECM, E), and polydeoxyribonucleotide (PDRN, P).	Polylactic glycolic acid, magnesium hydroxide, and bone ECM.	PME and PMEP groups displayed significantly increased biocompatibility compared to the PLGA group, and both scaffolds had an increased population of calcein-AM positive human bone-marrow mesenchymal stem cells (hBMSCs), i.e., live cells at one, three, and seven days post-implantation [117].
	ECM functionalized onto the surface of multi-channel biphasic calcium phosphate granules (MCG), seeded with MC3T3-E1 cells, and implanted into a rabbit femoral head defect model.	ECM functionalized onto the surface of multi-channel biphasic calcium phosphate granules (MCG).	Protein adsorption and osteogenic properties were improved on ECM functionalized MCG scaffolds compared to controls. ECM functionalized scaffolds enhanced bone regeneration in a rabbit model of a femoral head defect [118].
	Bone ECM was configured into demineralized bone paper (DBP) as a material to direct osteoblasts to deposit structural mineralized bone tissue, and it was seeded with osteoblasts from DsRed reporter mice.	ECM configured into demineralized bone paper (DBP).	DBP effectively stimulated the trabecular osteoid, directed rapid and structural mineralization by osteoblasts, and contained the microenvironment necessary to support bone remodeling. Compared to control cells cultured on TCP, cells grown in the DBP displayed significantly higher mineralization, collagen alignment, and elongated morphology that was aligned with the underlying lamellar structure of the demineralized bone [119].
	Osteoporotic mice models (established by ovariectomy) with three-dimensional complexes of encapsulated bone marrow derived mesenchymal stem cells (BMMSCs) in methacrylate gelatin (GelMA) hydrogels, and they inserted it into surgically induced femoral defects.	Methacrylate gelatin (GelMA) hydrogels.	Mitochondrial phosphoenolpyruvate carboxykinase (PCK2) promoted osteogenesis in three-dimensional ECM with tunable stiffness in vitro and in vivo. PCK2 enhanced the rate-limiting metabolic enzyme pallet isoform phosphofructokinase (PFKP) in three-dimensional ECM, and it further activated AKT/extracellular signal-regulated kinase 1/2 (ERK1/2) cascades to regulate osteogenic differentiation of MSCs [121].
	ECM-loaded three-dimensional printed gelatin (Gel), sodium alginate (SA), and 58s bioglass (58sBG) gels were seeded with either rat aortic endothelial cells (RAOECs) or rat bone mesenchymal stem cells (RBMSCs), and they were implanted into rat mandibular defects.	ECM-loaded three-dimensional printed gelatin (Gel), sodium alginate (SA), and 58s bioglass (58sBG) gels.	Scaffolds coated with ECM significantly increased the expression of osteogenic and angiogenic genes. ECM-scaffolds promoted bone defect healing in vivo compared to the pure scaffold. RAOECs–ECM scaffolds and RBMSCs–ECM scaffolds enhanced bone formation, likely via increased expression of RUNX2, OCN, BMP2, CD31, and VEGF [125].

## 3. Future Directions

Enhanced understanding of micro- and nanoscale mechanical properties of engineered ECM materials are considered here. 

Significant research has already been conducted to understand the mechanics and topography of ECM, as well as how these properties relate to cellular changes at a macroscopic level [126,127,128,129]. There is much opportunity to improve understanding of the ECM at the microscopic and nanoscale levels, which are the scales encountered by developing, differentiating, injured, and healing cells. Assessing these properties at the micro- and nano- length scales is critical, as several types of ECM exhibit mesh-like nanoscale structures with fiber diameter and pore size governing the topographical landscape [129]. Although there is conflicting, tissue-dependent data, most research has concluded that ECM scaffolding with pore sizes between 100 and 500 μm is optimal for cellular proliferation [130,131]. The fiber diameters in ECM specifically can range from 2 nm (fibronectin) to 500 nm (collagen) [132]. In order for the engineering of both natural and synthetic scaffolds to progress, the study of in vivo ECM at the micro and nanoscale levels is crucial for the accurate replication of feature height, porosity, fiber diameter, and mechanical properties of native ECM in engineered scaffolds and other biomimetic materials.

Atomic force microscopy (AFM) is a promising imaging modality for the study of the topography, mechanical properties, and other ECM characteristics at the micro- and nanoscopic levels. AFM is capable of precisely resolving the nano-topography of diverse surfaces and mapping the spatial distribution of their physicochemical properties to include charge density and potential in a variety of hard and soft materials, including hydrogels [133,134]. This knowledge is invaluable, as it associates surface charge with protein and cellular adhesion and interactions [135,136,137]. Furthermore, cells cultured in hydrogels frequently remodel their local environment, especially when immune cells are present as is the case for many disease model co-cultures, and these localized mechanical alterations are lost in macroscale mechanical testing [138].

AFM can quantify the stiffness of natural ECM and synthesized ECM scaffolds, which allows for correlations between the nanomechanical characteristics of surfaces and their impact on cellular interactions and behaviors. Cell behavior can then be compared to what has been observed in the context of the material’s bulk characteristics. AFM also offers a method of mechanical testing that is less destructive than standard techniques (i.e., tensile testing and microhardness). AFM can probe multiple mechanical properties simultaneously to produce accurate measurements that include stiffness, maximum indentation, indentation at maximum force, hysteresis, hardness, compliance, adhesion force, detachment distance, detachment energy, and Young’s modulus. As precise attributes of the ECM are engineered to elicit specific responses from cells or create individualized patient disease models, techniques, such as AFM, may prove invaluable in determining the exact properties of various ECM scaffolds.

While AFM stands as an incredibly powerful tool to analyze ECM at the microscopic and nanoscopic level, several experimental limitations remain. The long imaging time required for a high-resolution image, potential damages exerted by the probe tip, imaging artifacts, and quantitative interpretation of the results have constrained AFM’s utility in tissue engineering research. However, AFM has already been successfully used before to obtain quality concerning engineered graft surfaces [139]. This same study showed that loading type significantly influenced mechanical and histological outcomes of engineered cartilage surfaces, particularly in regards to the coefficient of friction at multiple length scales. This type of AFM-based analysis can indicate which types of loading may favor regeneration versus maintenance versus injury in cartilage [139]. It is within reason to expect similar studies in other musculoskeletal tissues to yield equally insightful results. As the demand for highly accurate disease models increases, we foresee increased use for AFM and similar techniques that can offer insight into the multiscale mechanical properties of engineered scaffolds, particularly those that incorporate biological components, such as ECM.

### 3.1. Genetically Engineered ECM and ECM-Based Bioinks

In addition to using highly sensitive mechanical testing, such as AFM, to inform engineered ECM and biomaterial scaffold design, we expect the role of genetic engineering to expand into the fabrication of custom, genetically-modified ECM for use in biomaterial scaffolds. As highlighted extensively in this review, a critical role of ECM is mechanotransduction. It stands to reason that engineered ECM from genetically modified cells/organisms that contains a specific array of mechanotransduction proteins may be a useful and desirable component of newly developed scaffolds. Techniques, such as CRISPR-based genome editing, may support the fabrication of custom ECMs that can replicate features of diseases that, until now, have been difficult or impossible to mimic in vitro. Alongside genetic engineering, novel composite materials, such as ECM-silk and ECM-titanium constructs, and ECM-based bioinks [78,80,101,140], may provide platforms with previously unachievable mechanical, biochemical, and genetic properties for use in regenerative therapies. Bioinks in particular are seeing increases in popularity and potential applications.

Until recently, most printers were unable to produce the slightly heterogeneous morphologies observed in native tissues. Recent gains in printer accuracy, porosity, and random structure generation are supporting the fabrication of biomaterials that mimic the native structure of small soft tissues, including musculoskeletal tissues. While ink availability has limited the application of this technology, new inks are rapidly emerging and enable multilayer printing while also incorporating growth factors, such as TGFβs and BMPs [100,141]. Advances in the rheological properties and printability of ECM-based materials via addition of methacrylation reactions or components continue to improve the usability of custom and commercial bioinks targeted for musculoskeletal tissue engineering applications [98]. Incorporating ECM into these inks may provide the missing cues needed to drive in situ tissue regeneration. These printed ECM and growth factor-augmented materials may seamlessly integrate with the patient’s own tissues and be further modifiable post-implantation, degradable via the body’s immune cells [142], and highly bio- and cytocompatible. We expect a prominent role for ECM as a component of the bioinks and biomaterials of the future.

### 3.2. Engineered ECM for Understanding and Treating Musculoskeletal Fibrosis

An underexplored application of engineered ECM is in understanding fibrotic disease, both in regards to musculoskeletal tissues and in the rest of the body. Significant interest in controlling ECM mechanics, and particularly ECM stiffening, has emerged in research related to idiopathic pulmonary fibrosis (IPF). IPF is a progressive scarring disease characterized by extracellular matrix accumulation and altered mechanical properties of lung tissue. Recent studies support the hypothesis that these compositional and mechanical changes create a progressive feed-forward loop, in which enhanced matrix deposition and tissue stiffening contribute to fibroblast and myofibroblast differentiation and activation, which further perpetuates matrix production and stiffening [143]. As the mechanotransduction pathways that sense and respond to the biomechanical properties of tissues are present across tissue types, work in one disease model may yield useful information to others. The relationship between stiffening and disease observed in IPF can be extended to musculoskeletal tissues, and indeed emerging evidence increasingly implicates localized ECM stiffening to disease initiation in the cartilage [45,50], tendons [144], and muscles [145,146,147]. Despite recent data, few studies have focused on musculoskeletal fibrosis specifically, largely due to the lack of ECM and ECM-composite materials that can accurately recreate the mechanical profile of fibrotic tissue.

Additional evidence of the critical role of matrix mechanics in fibrotic diseases comes from the field of vascular regeneration. Novel engineered ECM and polyethylene glycol- based materials were recently utilized to understand the pathophysiology of pulmonary artery hypertension (PAH), a progressive disease of the lung vasculature that is characterized by elevated pulmonary blood pressure, remodeling of the pulmonary arteries, and ultimately right ventricular failure. Therapeutic interventions for PAH are limited in part by the lack of in vitro screening platforms that accurately reproduce dynamic arterial wall mechanical properties. In this study, the ECM of the pulmonary arteries was engineered to mimic disease progression. The engineered ECM and polyethylene glycol-based model allowed for unprecedented recreation of the mechanical properties of both PAH and healthy tissue [148,149,150], providing a new in vitro tool for understanding how fibrotic pathogenesis initiates. The phototunable constructs developed to mimic the mechanical properties of normal and diseased arteries could be adapted for use in musculoskeletal applications, providing an existing and validated platform for interrogating the role of the ECM in musculoskeletal health and disease.

### 3.3. Limitations

As with any literature summary, we intend for this review to be a brief survey of each of these commonly studied tissues, rather than a comprehensive guide to every engineered ECM-based approach in the field of musculoskeletal regeneration. This review fills a gap in the existing literature by uniting a diverse array of experimental approaches under the common theme of musculoskeletal regeneration. While highlighting every recent and exciting use of engineered ECM-cell interactions in the context of musculoskeletal regeneration is outside of the scope of this review, and we show the expansive promise of ECM in musculoskeletal tissue engineering. We believe this grouping serves readers who may work in more than one musculoskeletal tissue system, which is an increasingly common theme in musculoskeletal tissue engineering.

## 4. Conclusions

Throughout the last decade, the expanding ability to direct cell behavior for musculoskeletal tissue regeneration via the use of engineered ECM and ECM-based materials has greatly expanded our knowledge of musculoskeletal development, disease, and healing. This review has focused on recent and noteworthy advances that incorporate cell, ECM, and material interactions to increase the regenerative capacity of muscle, cartilage, bone, and tendon. We examined approaches focused on a single tissue, as well as studies that incorporated two or more musculoskeletal tissues, to more faithfully recreate the in vivo environment. The works we highlight suggest that single- and multi-tissue approaches can generate valuable insights into how the physical, mechanical, and biochemical attributes of the ECM, alone and in combination with innovative engineered materials of natural and synthetic origins, can be harnessed to direct cellular behavior towards maximizing the regenerative capacity of musculoskeletal tissues. Human and animal ECM both deserve continued exploration, as continuous improvements to decellularization techniques reduce the danger of systemic immune response or rejection.

We concluded with predictions of the future of engineered cell–ECM–material interactions. The implementation of advanced manufacturing techniques, such as three-dimensional printing, as well as improvements in decellularization protocols that decrease the likelihood of adverse immune reactions, have paved the way for expanded use of ECM in musculoskeletal tissue engineering. Renewed interest in harnessing the ECM both as a standalone material and in combination with other well-established biomaterials is driven by studies showing that ECM instructs cells to behave more closely to how they do in native tissue. We show the expansive promise of ECM in musculoskeletal tissue engineering, and we look forward to the advancements of the coming decade. Ultimately, we expect meaningful and exciting advances in bioprinting, improved decellularization techniques, genetic engineering of both the ECM and cells, and the further determination of how ECM mechanics at multiple length scales can be exploited to precisely direct cell behavior towards regenerative phenotypes.

## Data Availability

No new data were created or analyzed in this study. Data sharing is not applicable to this article.
